# *C. acnes* qPCR-Based Antibiotics Resistance Assay (ACQUIRE) Reveals Widespread Macrolide Resistance in Acne Patients and Can Eliminate Macrolide Misuse in Acne Treatment

**DOI:** 10.3389/fpubh.2022.787299

**Published:** 2022-03-18

**Authors:** Jingheng Zhang, Fang Yu, Keyun Fu, Xinyu Ma, Yi Han, Chi Ching Ali, Haonan Zhou, Yantao Xu, Tingyue Zhang, Shuntong Kang, Yiming Xu, Zhuolin Li, Jiaqi Shi, Shuai Gao, Yongyi Chen, Liyu Chen, Jianglin Zhang, Feizhou Zhu

**Affiliations:** ^1^Xiangya School of Medicine, Central South University, Changsha, China; ^2^Department of Biochemistry and Molecular Biology, School of Life Sciences, Central South University, Changsha, China; ^3^Department of Dermatology, Xiangya Hospital, Central South University, Changsha, China; ^4^Hunan Cancer Hospital, The Affiliated Cancer Hospital of Xiangya School of Medicine, Central South University, Changsha, China; ^5^Clinical Research Center for Wound Healing in Hunan Province, Changsha, China; ^6^Department of Microbiology, School of Basic Medical Sciences, Central South University, Changsha, China; ^7^Department of Dermatology, Shenzhen People's Hospital, Shenzhen, China; ^8^The Second Clinical Medical College, Jinan University, Guangzhou, China; ^9^The First Affiliated Hospital, Southern University of Science and Technology, Shenzhen, China; ^10^Hunan Key Laboratory of Animal Models for Human Diseases, Changsha, China

**Keywords:** acne (acne vulgaris), antimicrobial resistance, quantitative PCR (qPCR), antimicrobial, macrolide-resistant gene

## Abstract

**Background:**

Macrolides have been widely used to treat moderate-to-severe acne for more than 50 years. However, the prevalent antibiotic resistance of Propionibacterium acnes, along with the absence of clinically available resistance tests, has made macrolide misuse a frequent occurrence around the globe, with serious consequences.

**Objective:**

We developed *Cutibacterium acnes quantitative PCR* (qPCR)-based antibiotics resistance assay (ACQUIRE) to enable fast and accurate detection of *C. acnes* macrolide resistance in clinical settings, representing an opportunity to administer antibiotics more wisely and improve the quality of care.

**Methods:**

A cross-sectional observational study (*n* = 915) was conducted to probe into the macrolide resistance of *C. acnes* in patients with acne.

**Results:**

The high sensitivity of ACQUIRE enabled us to reveal a much higher *C. acnes* 23S recombinant DNA (rDNA) point mutation rate (52%) and thus a higher macrolide resistance (75.5%) compared to previous reports. Carriage of ermX gene was discovered on 472 (53%) subjects, which concurs with previous studies.

**Conclusion:**

The macrolide resistance of *C. acnes* is much higher than previously reported. Integrating ACQUIRE into acne treatment modalities may eliminate macrolide misuse and achieve better clinical improvements.

## Introduction

Acne vulgaris, one of the most common skin disorders worldwide, is inflicting billions of patients, significantly impairs their quality of life. The pathogenesis of acne involves the release of inflammatory mediators, skin hyper-keratinization, increased sebum secretion, and colonization of *Propionibacterium acnes* (recently reclassified as *Cutibacterium acne*s) (1–5). As the dominant commensal of the human pilosebaceous unit, *C. acnes* is intimately involved in the development of acne. It not only disturbs the proliferation of keratinocytes, but also carries lipase, protease, and hyaluronidase activity which can damage the pilosebaceous unit and induce inflammation (6). *C. acnes* can also activate toll-like receptors (7) and protease-activated receptors expressed by keratinocytes, which in turn induces the production of interleukins and matrix metalloproteinases (8).

Antibiotics have been used in acne treatment for more than 50 years, representing an essential part of the first-line treatment for moderate-to-severe acne (9). Macrolides (erythromycin, clarithromycin, and azithromycin) and tetracyclines (minocycline and doxycycline) are two classes of antibiotics that are frequently applied (1, 10). However, the extensive use of macrolides has caused escalating *C. acnes* resistance worldwide. Increases in *C. acnes* resistance have now been reported in all major regions within China and around the globe (11–20), with many countries reporting more than 50% of macrolide-resistant *C. acnes* (21). If macrolide is prescribed, the carriage of macrolide-resistant *C. acnes* strains may cause reduced treatment response, relapse, or prolonged course of disease for acne patients, increasing the disease burden of acne.

Although *C. acnes* exhibit far less resistance to tetracyclines (21), compared with macrolide, tetracyclines have several shortcomings, including more common adverse effects, potential liver and kidney toxicity, and the incompatibility with oral isotretinoin. Hence macrolide remains essential in acne management in China and many other countries. Also, simply abolishing macrolide and initiating the extensive use of tetracyclines will result in the rapid escalation of tetracycline resistance and cause waste of antibiotic resources that are insufficient already. Therefore, the most desirable practice for dermatologists would be prescribing tetracyclines to patients who carry macrolide-resistant *C. acnes*, and administering macrolide to those whose *C. acnes* is susceptible to macrolide.

However, the current antimicrobial susceptibility test (AST) of *C. acnes* employs a series of time-, labor-, and cost-intensive procedures due to the nature of *C. acnes*. As an anaerobe, *C. acnes* require a special culture environment and propagate slowly, so the test result could only be available after a week post sampling. The sensitivity of the current culture-based method is also unsatisfactory because the resistant *C. acnes* would be hard or even impossible to isolate if existing only in a low-percentage, which is often the case when the mutation occurred naturally but had not undergone antibiotic selection. Those drawbacks not only prevent any chance of applying the *C. acnes* resistance test clinically, but also impose huge restrictions on related scientific research.

Toward those ends, we developed *C. acnes* quantitative PCR (qPCR)-based antibiotics resistance assay (ACQUIRE), a method that utilizes the comedones (whitehead or blackhead) extracted from patients' follicles based on ARMS-qPCR (Amplification refractory mutation system qPCR), and is capable of determining the presence of macrolide-resistant *C. acnes* within 3 h, showing great potential to serve as a routine test for patients with clinical moderate-to-severe acne. ARMS-qPCR is widely used as a convenient and cost-saving tool to detect the point mutation and SNPs in nucleic acids (22). However, it has not been utilized to determine the resistance determinants in *C. acnes* before this study.

With ACQUIRE, we conducted a large-scale cross-sectional observational study to examine the macrolide resistance level of *C. acnes*.

## Methods

### Participants and Study Design

**Figure 1:**
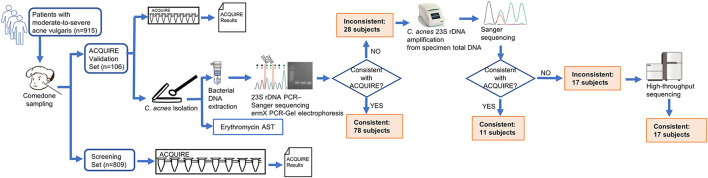
Schematic diagram of study design. The patients enrolled were sampled and randomly assigned into two sets. For patients in the *Cutibacterium acnes* quantitative PCR (qPCR)-based antibiotics resistance assay (ACQUIRE) Validation Set whose ACQUIRE results and result of culture method were inconsistent, the *C. acnes* 23S recombinant DNA (rDNA) was amplified from specimen total DNA and subject to Sanger sequencing. If the ACQUIRE results and the result of Sanger sequencing were still inconsistent, the amplification product was subject to high-throughput sequencing (Illumina Miseq), sequence filtration (sequences with <95% similarity to *C. acnes* were discarded), and mutation rate analysis.

A total of 915 moderate-to-severe acne patients were enrolled from December 2017 to January 2020 at both the Department of Dermatology, Xiangya Hospital, Central South University, and a skincare institution (Miao'miao-qingfu) to ensure a wide spectrum of enrolled patients. The inclusion criteria were: (1) 12–50 years of age; (2) moderate-to-severe acne, classified by the global acne grading system (GAGS) with a score ≥ 19. The exclusion criteria were: (1) history of systemic acne treatment within 6 months; (2) mild acne (GAGS score < 19). All patients provided informed consent and the study was approved by the ethics committee of Xiangya Hospital. Enrolled patients were all sampled and randomly assigned into two non-intersecting sets (Figure 1).

To the ACQUIRE Validation Set (*n* = 106), acne lesions from multiple sites of the subjects' faces were squeezed using a sterile comedone extractor to obtain the comedone specimens (whitehead or blackhead). The specimens were then tested by ACQUIRE. A small portion of the specimens was reserved for *C. acnes* isolation in order to compare the results of ACQUIRE and the current method. To the Screening Set (*n* = 809), the specimens were also obtained and tested.

Specimens were obtained from 915 subjects (525 women and 390 men) and were tested by ACQUIRE. Subjects were 12–49 years of age (mean ± SD, 24.1 ± 5.7 years). Among 915 subjects, age and sex distributions among 12 genotypes were not significantly different.

### *C. acnes* Isolation, Identification, Genotyping, and Antimicrobial Susceptibility Testing

Isolation, identification, 23S rDNA mutation, ermX detection, and AST of *C. acnes* were adopted from previous studies (23). Briefly, *C. acnes* were isolated on CDC anaerobic blood agar under an anaerobic atmosphere and at 37°C. The 16S recombinant RNA (rRNA) gene of isolated bacteria was amplified and sequenced to identify *C. acnes* from isolated strains. The ermX carriage status and the genotype of 23S rDNA of isolated *C. acnes* were determined by PCR -agarose gel electrophoresis and PCR-sanger sequencing, respectively. The minimum inhibitory concentration (MIC) of *C. acnes* to erythromycin was measured by broth microdilution. Brucella Broth with serial erythromycin concentration (0, 0.25, 0.5, 1, 2, 4, 8, 16, 32, 64, 128, and 256 ug/mL) was dispensed into 96-well plate, and strain suspension with a density of 0.5 Mcfarland Standard was inoculated. The plate was anaerobically incubated for 72 h and OD600 was measured with a plate reader (EnSpire 2300, Perkin Elmer). An MIC90 above or equal to 1 μg/ml was considered resistant. The anaerobic atmosphere in this study was generated by putting an Anaeropack sachet (C-1, Mitsubishi Gas Chemical) into an air-tight culture box, and was inspected by observing the color of an oxygen indicator (C-22, Mitsubishi Gas Chemical).

### The Development and Application of ACQUIRE

Mechanistically*, C. acnes* gains macrolide resistance through the presence of the ermX gene in its genome and 23S rDNA point mutation. The ermX gene, which often is carried by a corynebacterial-origin transposon Tn5432, makes *C. acnes* exhibit constitutive MLS_B_ (macrolide-lincosamine-type B streptogramin) resistance (24). Point mutations in 23S rDNA at *Escherichia coli*-equivalent bases 2,058(A>T, A>G) and 2,059(A>G), which affect the peptidyl transferase region of the ribosome, makes *C. acnes* exhibit constitutive MLS_B_ resistance and macrolide-only resistance, respectively (25).

As the dominant organism of skin follicle microbiota, *C. acnes* takes up ~50–90% of organisms in skin follicles (26, 27), which is a huge advantage of utilizing comedone as the test specimen. To eliminate the interference caused by the DNA of other organisms, primers were designed against the sites that showed the greatest inter-species variation. Sequences of ACQUIRE primers and probes were:

C.acnes-23S-F: GGGGACCTGAGCTGTC;
ARMS-58A-R: CTATAGTAAAGGTCCCGGGGTCGYT;
ARMS-58T-R: CTATAGTAAAGGTCCCGGGGTCGYA;
ARMS-58G-F: CTATAGTAAAGGTCCCGGGGTCGYC;
ARMS-59A-R: CTATAGTAAAGGTCCCGGGGTCAT;
ARMS-59G-R: CTATAGTAAAGGTCCCGGGGTCAC;
23S-probe: FAM-CATCTTTACTCGTAATGCAATTTCACC-BHQ1;
ermX-F: GATGATGACGGCTCAGTGGT;
ermX-R: TTTGCGCTGCTCTATCGGAA;
ermX-probe: FAM-TTCACATTTCACCTGGGTTCTCGGGTACCAA-BHQ1; C.acnes-16S-F: ACCGCTTTCGCCTGTGAC;
C.acnes-16S-R: AGCCCC AAGATTACACTTCCGAC;
16S-probe: VIC-AAGCGTGAGTGACGGTAATGGGTAAAGAAGCACC-BHQ1.

**Figure 2:**
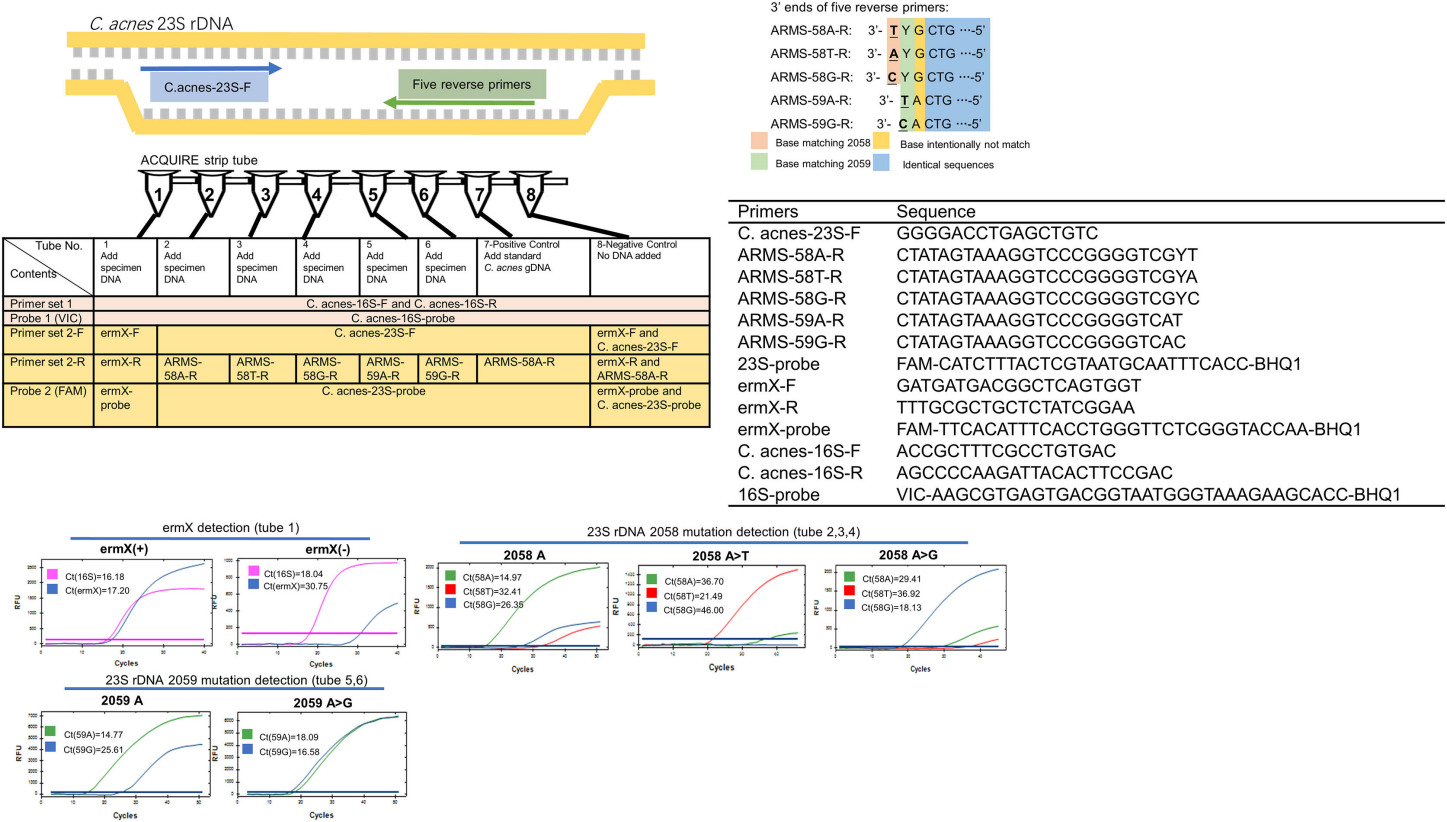
Working principle, composition, and typical results of ACQUIRE. Composed of 10 PCR primers and three fluorescence probes, ACQUIRE is integrated into one 8-strip tube. Tube 1 interrogates ermX presence, tubes 2, 3, and 4 discriminate 2,058 A>T and 2,058 A>G from 2,058A. Tubes 5 and 6 discriminate 2,059 A>G from 2,059A. Tube 7 and 8 serve as a positive and negative control, respectively. The discrimination of 23S rDNA mutations was achieved by the Ct value difference caused by the match or mismatch of 3' ends of five reverse primers. Additional mutations (base intentionally not match) were introduced into primers to strengthen this effect.

Once the specimen was obtained, it was transferred into a 1.5 ml microcentrifuge tube prefilled with 175 μl lysis buffer (Cat. 939016, Qiagen) and glass beads (40 mesh, 0.425 mm in diameter). Then, the microcentrifuge tube was fixed on a tissue disruptor (4,000 rpm, 10 min) to release the *C. acnes* that resided inside the specimen. Then, the total bacterial DNA was extracted using a universal Gram-positive bacteria DNA extraction kit (Cat. FP211, Tiangen Biotech, China), and dissolved in 100 μl sterile TE buffer (Cat. 17000-10, Qiagen). The eluted DNA was loaded into an ACQUIRE strip tube (0.5 μL eluted DNA per tube) prefilled with primers, probes, water, and a qPCR reaction mix. Then, the qPCR protocol was run and the raw results were analyzed and the assay result was determined accordingly (Figure 2).

### Statistical Analysis

Statistical analysis was performed on SPSS 22.0 software (IBM, Armonk, NY, USA). Continuous variables were expressed as mean ± SD and compared using the Mann-Whitney *U*-test for two groups. Categorical variables were expressed as frequencies and percentages and compared using the chi-squared test (*n* > 5) or Fisher's exact test (*n* < 5). For all statistical analyses, a 2-sided *P* < 0.05 was accepted as statistically significant.

## Results

### ACQUIRE Results Were as Accurate as Sequencing Method, but Cost Much Less Time

**Figure 3:**
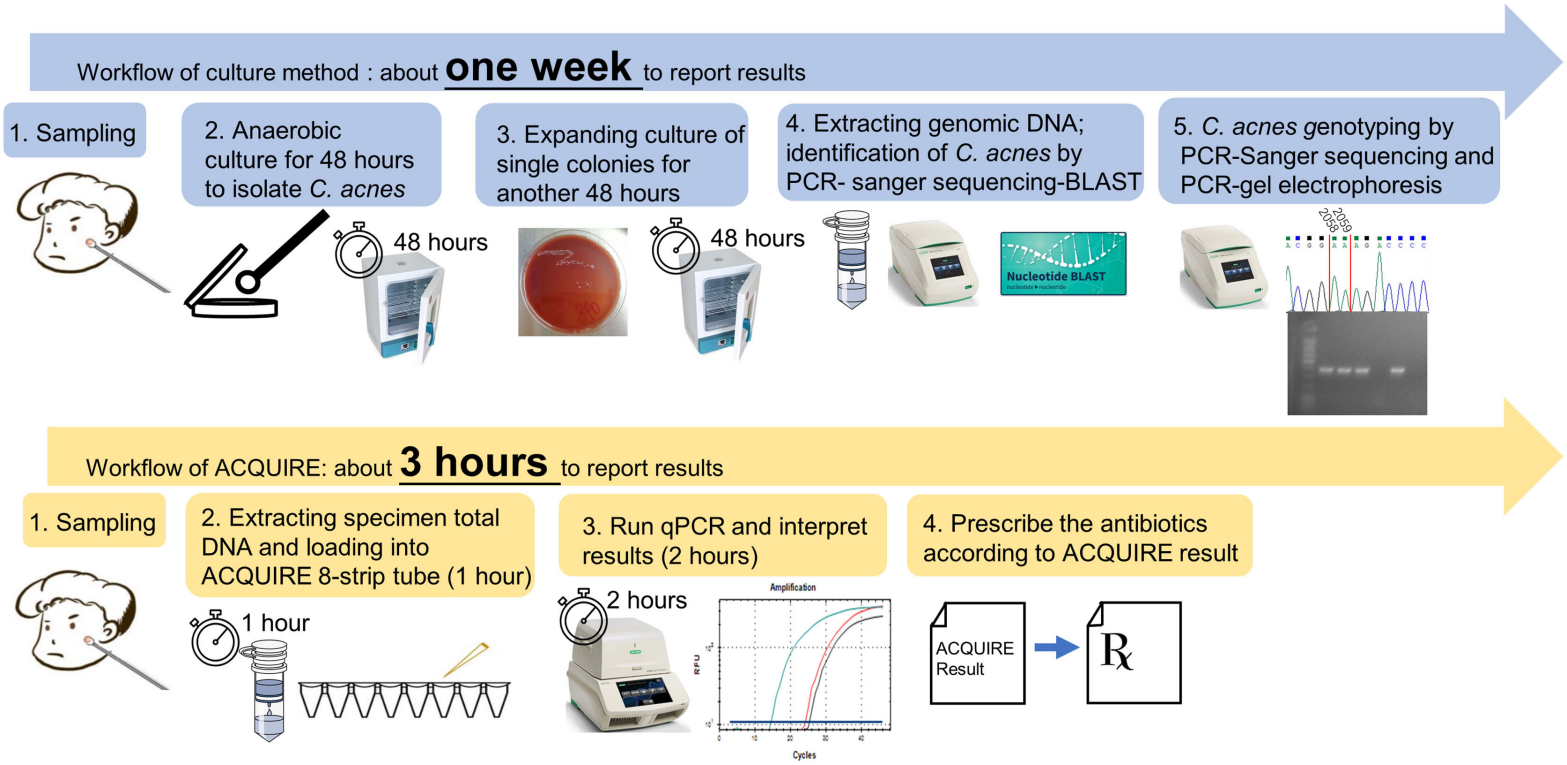
Comparison of the workflow and time-cost between the current method and proposed ACQUIRE.

Among 106 subjects, the results of ACQUIRE and validation were not significantly different concerning the macrolide susceptibility phenotype (*P* = 0.377) (Table 1). In the ACQUIRE results, 31 (29.2%) subjects were macrolide-susceptible genotype and 75 (70.8%) were macrolide-resistant genotype. In the validation result, consistent with the ACQUIRE results, 37 (34.9%) subjects were macrolide-susceptible genotype and 69 (65.1%) were macrolide-resistant genotype (Table 1). Compared with ACQUIRE, the PCR+Sequencing method, which is culture-dependent, costs much more time (Figure 3). The detailed results of the ACQUIRE validation process were recorded in Supplementary Table 1.

**Table 1:** ACQUIRE is a sensitive and accurate method for detecting *P. acnes* resistance.

**Phenotype**	**Genotype** **of specimen**		**ACQUIRE Validation Set**					**ACQUIRE result of** **all subjects (*n* = 915)**
			**ACQUIRE** **result** **(*n* = 106)**	**Validation** **result** **(*n* = 106)^**b**^**	**AST of** ***C. acnes*** **isolates (*****n*** **=** **123)**^**c**^		
					**S**	**R**	**Total**	
Macrolide susceptible	AA	ermX (-)	31 (29.2%)	37 (34.9%)	62	0	62 (50.4%)	224 (24.5%)
Macrolide resistant	AA	ermX (+)	31 (29.2%)	25 (23.5%)	3	36	61 (49.6%)	207 (22.6%)
	TA	ermX (+)	8 (7.5%)	7 (6.6%)	0	3		66 (7.2%)
		ermX (-)	11 (10.4%)	12 (11.3%)	0	7		42 (4.6%)
	GA	ermX (+)	4 (3.8%)	4 (3.8%)	0	2		81 (8.9%)
		ermX (-)	1 (0.9%)	1 (0.9%)	0	3		50 (5.5%)
	AG	ermX (+)	8 (7.5%)	8 (7.5%)	0	1		87 (9.5%)
		ermX (-)	5 (4.7%)	5 (4.7%)	0	6		73 (8.0%)
	GG	ermX (+)	0 (0.0%)	0 (0.0%)	0	0		27 (3.0%)
		ermX (-)	3 (2.8%)	3 (2.8%)	0	0		22 (2.4%)
	TG	ermX (+)	2 (1.9%)	1 (0.9%)	0	0		22 (2.4%)
		ermX (-)	2 (1.9%)	3 (2.8%)	0	0		14 (1.5%)
		Total	75 (70.8%)	69 (65.1%)	65 (52.8%)	58 (47.2%)		691 (75.5%)
*P*-value				0.377^a, d^		0.702^a, e^		

*AST, antimicrobial susceptibility test; S, susceptible; R, resistant*.

a *Chi-squared test*.

b *Validation result of one specimen refers to the combination of (1) genotypes of isolated C. acnes and (2) result of Sanger sequencing or high-throughput sequencing of the 23S rDNA amplicon of specimen*.

c *AST was conducted with isolated C. acnes from the specimen*.

d *Results of ACQUIRE and validation results were analyzed, and no significant difference were found*.

e *Genotypes [AA, ermx (-) and the rest] and phenotypes (macrolide-susceptible and macrolide-resistance) of isolated C. acnes were analyzed, and no significant difference were found*.

### Erythromycin Susceptibility Was Consistent With Genotypes of *C. acnes*

A total of 123 *C. acnes* strains were isolated from 106 subjects, and their genotype and susceptibility to erythromycin were determined. Consistent with previous studies, the genotype of *C. acnes* well-correlated with their phenotype of macrolide susceptibility (*P* = 0.702), with only three inconsistent strains (Table 1).

### ACQUIRE Revealed a Higher Macrolide Resistance Rate Due to More Prevalent 23S rDNA Mutation

Among 915 subjects whose specimen was tested by ACQUIRE, ermX was the most frequently detected macrolide-resistance determinant and was discovered on 472 (53%) subjects, which concurs with previous studies (21). However, the prevalence of 23S rDNA mutation is much higher than previous reports, with 468 (52%) subjects carrying at least one mutation. The proportion of macrolide-resistant subjects was also higher, with 691 (75.5%) being resistant to macrolide (Table 1).

## Discussion

The increasing *C. acnes* resistance asks for dermatologists to utilize those antibiotics that are still effective, which is a limited and diminishing clinical asset, more prudently and smartly. However, due to the inability of the current culture-based method, the status quo of *C. acnes* resistance is underestimated, and the related clinical and microbiological studies are insufficient.

Of note, simply switching the oral antibiotic to tetracyclines is not the once-and-for-all solution for *C. acnes* resistance and associated treatment failure, since resistant mutations for tetracyclines can also emerge in *C. acnes* and accumulate in the population.

Over the last 20 years, multiple studies (12, 13, 18, 23–25) have well-established and confirmed the correlation between the *C. acnes* genotype (23S rDNA 2058_2059 mutation and ermX carriage status) and its macrolide susceptibility phenotype, which laid the foundation for developing a macrolide resistance assay for *C. acnes* based on its genotype. However, the molecular basis of *C. acnes* resistance to tetracyclines has not been well-elucidated, which regrettably made it impossible to integrate the tetracycline resistance detection of *C. acnes* into ACQUIRE.

As a supplement to the current acne treatment algorithm, ACQUIRE can be used whenever a macrolide agent is intended to be prescribed, including topical and oral agents, and leave the rest of treatment options uninfluenced, which made it easy to integrate ACQUIRE into current treatment modalities and guidelines. ACQUIRE also provides an easy, scalable, and economical way to probe into the *C. acnes* resistance status in a large population.

By eliminating antibiotic misuse, ACQUIRE can make acne treatment more effective with less cost and shortened course of the disease, representing an opportunity to improve the quality of care. In this proof-of-concept study, we demonstrated the possibility and efficacy of integrating ACQUIRE into acne treatment modalities and achieved a better clinical outcome. However, large-scale multi-center randomized controlled trials are urgently needed to provide more solid evidence.

In the future, with advancements in the field, functions of ACQUIRE can be further extended, for example, by adding more resistance determinants into the current assay panel to enable the antibiotic resistance detection of tetracyclines and other antibiotics, or enabling the discrimination and quantification of different *C. acnes* subgroups, or probing into the carriage status of acne susceptible genes in the genome of the patient.

Meanwhile, general principles to slow the development of antibiotic resistance should always be encouraged, like using Benzoyl Peroxide (BPO), strictly limiting long-term (more than 2 months) antibiotic usage (28), and adopting alternative therapies like laser and light-based treatments (29).

## Conclusion

The macrolide resistance of *C. acnes* is higher than previously reported due to more prevalent 23S rDNA mutations. Future studies should focus on the efficacy of treatment guided by ACQUIRE in large-scale cohorts.

## Data Availability Statement

The high-throughput sequencing data involved in this article has been deposited in NCBI SRA database under the accession number PRJNA768297 and is made publically accessible with the following link: https://www.ncbi.nlm.nih.gov/sra/PRJNA768297.

## Ethics Statement

The studies involving human participants were reviewed and approved by Ethics Committee of Xiangya Hosiptal, Central South University. The patients/participants provided their written informed consent to participate in this study.

## Author Contributions

JHZ designed the study, conducted the experiments, and drafted the manuscript. FY conducted half of the qPCR experiment and collected patient demographic data. KYF, XYM, YH, CCA, HNZ, and YMX processed clinical specimen and isolated bacterial strains. TYZ, YTX, and STK provided valuable opinion, analyzed data, and revised the manuscript. ZLL, JQS, SG, and YYC provided conceptual insights and conducted microbiology experiments. LYC, JLZ, and FZZ participated in the study design, manuscript revision, acquired funding, and supervised the research. All authors contributed to this manuscript by drafting or revising the manuscript and approved the submitted version.

## Funding

This study was supported by China Hunan Provincial Science and Technology Plan (2018SK7005 and 2017SK2092), Innovative Education Reform Program of Central South University (2019CG045), National Undergraduate Innovation Training Program of Central South University (201810533233 and GS201910533150X), and Hunan Science and Technology Innovation Talent Program (to YMX).

## Conflict of Interest

FZZ, JLZ, LYC, JHZ, and FY reported China Patent 2020101633011, a method that promotes the detection accuracy and efficiency of *C. acnes* antibiotic resistance. The remaining authors declare that the research was conducted in the absence of any commercial or financial relationships that could be construed as a potential conflict of interest.

## Publisher's Note

All claims expressed in this article are solely those of the authors and do not necessarily represent those of their affiliated organizations, or those of the publisher, the editors and the reviewers. Any product that may be evaluated in this article, or claim that may be made by its manufacturer, is not guaranteed or endorsed by the publisher.
