# Supplementary material for: C. acnes qPCR-Based Antibiotics Resistance Assay (ACQUIRE) Reveals Widespread Macrolide Resistance in Acne Patients and Can Eliminate Macrolide Misuse in Acne Treatment
Source: Front Public Health. 2022 Mar 18;10:787299. doi: 10.3389/fpubh.2022.787299 (PMC8971513; doi:10.3389/fpubh.2022.787299)
Supplement: Supplementary file 1 [file Data_Sheet_1.PDF]

patient ID	ACQUIRE result		C. acnes strain ID	Strain phenotype			Strain genotype		Need furthur validation?	*C. acnes* 23S rDNA PCR of specimen	
	ermx status	2058_2059 genotype		MIC50	MIC90	Suscepti-bility	ermX PCR result	2058_2059 PCR-sequencing		Amplicon Sanger Sequencing	Amplicon High-Throughput Sequencing
1	+	AA	1-1	≥256	≥256	R	+	AA			
2	-	TG	2-1	≥256	≥256	R	-	AG	Yes	AG	A2058T, 6.07%; A2059G, 58.40%
3	-	TA	3-1	≤0.25	≤0.25	S	-	AA	Yes	AA	A2058T, 3.32%
4	+	AA	4-1	≥256	≥256	R	+	AA			
5	+	GA	5-1	≥256	≥256	R	+	AA	Yes	AA	A2058G, 33.54%
6	+	GA	6-1	≥256	≥256	R	+	GA			
7	+	TA	7-1	≥256	≥256	R	+	AA	Yes	AA	A2058T, 3.10%
8	-	TA	8-1	≥256	≥256	R	-	TA			
9	-	AA	9-1	0.25	0.5	S	-	AA			
10	+	AA	10-1	≥256	≥256	R	+	AA			
11	+	AA	11-1	0.25	0.5	S	+	AA			
12	-	AA	12-1	0.25	0.5	S	-	AA			
13	-	TA	13-1	≥256	≥256	R	-	TA			
14	+	AG	14-1	≥256	≥256	R	+	AA	Yes	AA	A2059G, 7.46%
15	-	TA	15-1	0.25	0.5	S	-	AA	Yes	AA	A2058T, 2.85%
16	-	AA	16-1	0.25	0.5	S	-	AA			
17	-	AG	17-1	0.25	0.5	S	-	AA	Yes	AA	A2059G, 8.00%
18	-	AG	18-1	≤0.25	≤0.25	S	-	AA	Yes	AG	
19	-	AA	19-1	0.25	0.5	S	-	AA			
20	-	AA	20-1	0.25	0.5	S	-	AA			
21	+	AA	21-1	0.25	0.5	S	+	AA			
22	+	AA	22-1	≥256	≥256	R	+	AA			
23	-	AA	23-1	≤0.25	≤0.25	S	-	AA			
24	-	TA	24-1	≤0.25	≤0.25	S	-	AA	Yes	TA	
25	-	AA	25-1	≤0.25	≤0.25	S	-	AA			
26	+	AA	26-1	≥256	≥256	R	+	AA			
27	+	AG	27-1	≥256	≥256	R	+	AG			
28	-	AA	28-1	0.25	0.5	S	-	AA			
29	-	GA	29-1	≤0.25	≤0.25	S	-	AA	Yes	AA	A2058G, 1.92%
30	+	TA	30-1	≥256	≥256	R	+	TA			

31	+	AA	31-1	≥256	≥256	R	+	AA			
32	+	AA	32-1	≥256	≥256	R	+	AA			
33	-	TA	33-1	64	128	R	-	TA			
34	+	AG	34-1	0.25	0.5	S	-	AA	Yes	AA	A2059G, 7.66%
			34-2	32	64	R	+	AA			
35	-	AA	35-1	0.25	0.5	S	-	AA			
36	+	AG	36-1	0.25	0.5	S	+	AA	Yes	AG	
37	+	GA	37-1	64	128	R	-	GA			
			37-2	64	128	R	+	GA			
38	-	AA	38-1	≤0.25	≤0.25	S	-	AA			
39	+	AA	39-1	0.25	0.5	S	-	AA			
			39-2	32	64	R	+	AA			
40	-	AA	40-1	0.25	0.5	S	-	AA			
41	+	AA	41-1	64	128	R	+	AA			
42	-	AA	42-1	0.25	0.5	S	-	AA			
43	-	TA	43-1	0.25	0.5	S	-	AA	Yes	AA	A2058T, 4.90%
44	-	TA	44-1	≥256	≥256	R	-	TA			
45	-	TA	45-1	0.25	0.5	S	-	AA	Yes	TA	
46	-	AA	46-1	0.25	0.5	S	-	AA			
47	-	GG	47-1	≥256	≥256	R	-	AG			
			47-2	0.25	0.5	S	-	AA			
			47-3	≥256	≥256	R	-	GA			
48	+	TA	48-1	≥256	≥256	R	+	TA			
49	-	AA	49-1	≤0.25	≤0.25	S	-	AA			
50	+	AA	50-1	≥256	≥256	R	+	AA			
			50-2	0.25	0.5	S	-	AA			
51	+	AA	51-1	16	32	R	+	AA			
52	+	AG	52-1	≥256	≥256	R	+	AA	Yes	AG	
53	+	AA	53-1	≥256	≥256	R	+	AA			
54	+	AA	54-1	≥256	≥256	R	+	AA			
55	+	AA	55-1	≥256	≥256	R	+	AA			
			55-2	≤0.25	≤0.25	S	-	AA			
56	-	GG	56-1	≥256	≥256	R	-	AG	Yes	AG	A2058G, 5.80%; A2059G, 58.13%
57	+	AA	57-1	0.25	0.5	S	-	AA			
58	+	GA	58-1	≥256	≥256	R	+	AA	Yes	AA	A2058G, 1.91%
			58-2	≤0.25	≤0.25	S	-	AA			

59	-	AA	59-1	0.25	0.5	S	-	AA			
60	-	AA	60-1	0.25	0.5	S	-	AA			
61	-	AA	61-1	≤0.25	≤0.25	S	-	AA			
62	-	AA	62-1	≤0.25	≤0.25	S	-	AA			
63	+	AA	63-1	≥256	≥256	R	+	AA			
			63-2	≤0.25	≤0.25	S	-	AA			
64	+	AA	64-1	≥256	≥256	R	+	AA			
65	+	AG	65-1	≥256	≥256	R	+	AA	Yes	AG	
			65-2	0.25	0.5	S	-	AA			
66	-	AA	66-1	≤0.25	≤0.25	S	-	AA			
67	-	AG	67-1	≥256	≥256	R	-	AG			
68	+	AA	68-1	≤0.25	≤0.25	S	-	AA			
69	+	AG	69-1	≥256	≥256	R	+	AA	Yes	AA	A2059G, 7.84%
70	+	AA	70-1	0.25	0.5	S	-	AA			
			70-2	≥256	≥256	R	+	AA			
71	+	AG	71-1	≥256	≥256	R	+	AA			
			71-2	≥256	≥256	R	-	AG			
72	-	AA	72-1	≤0.25	≤0.25	S	-	AA			
73	+	TG	73-1	≤0.25	≤0.25	S	-	AA	Yes	AA	A2058T, 7.18%; A2059G, 8.79%
74	+	AA	74-1	≤0.25	≤0.25	S	-	AA			
75	+	AA	75-1	≥256	≥256	R	+	AA			
76	+	TA	76-1	≥256	≥256	R	+	TA			
77	+	AA	77-1	0.25	0.5	S	-	AA			
78	-	AA	78-1	≤0.25	≤0.25	S	-	AA			
79	-	AA	79-1	0.25	0.5	S	-	AA			
80	+	TA	80-1	≥256	≥256	R	+	AA	Yes	TA	
			80-2	≤0.25	≤0.25	S	-	AA			
81	-	AA	81-1	≤0.25	≤0.25	S	-	AA			
82	+	AA	82-1	≥256	≥256	R	+	AA			
			82-2	0.25	0.5	S	-	AA			
83	-	AA	83-1	≤0.25	≤0.25	S	-	AA			
84	-	AA	84-1	≤0.25	≤0.25	S	-	AA			
85	-	AA	85-1	≤0.25	≤0.25	S	-	AA			
86	+	AA	86-1	≥256	≥256	R	+	AA			
87	-	AG	87-1	0.25	0.5	S	-	AA	Yes	AG	

88	+	AA	88-1	≤0.25	≤0.25	S	-	AA			
			88-2	≥256	≥256	R	+	AA			
89	-	AA	89-1	0.25	0.5	S	-	AA			
90	+	AA	90-1	0.25	0.5	S	-	AA			
91	-	TA	91-1	0.25	0.5	S	-	AA			
			91-2	≥256	≥256	R	-	TA			
92	+	AA	92-1	≥256	≥256	R	+	AA			
93	-	AA	93-1	0.25	0.5	S	-	AA			
94	-	TA	94-1	≥256	≥256	R	-	TA			
95	-	AA	95-1	0.25	0.5	S	-	AA			
96	+	TA	96-1	≥256	≥256	R	-	TA			
97	+	AA	97-1	≤0.25	≤0.25	S	-	AA			
98	+	AA	98-1	≥256	≥256	R	+	AA			
99	-	GG	99-1	≥256	≥256	R	-	GA	Yes	GG	
100	+	TG	100-1	16	32	R	-	AG	Yes	AG	A2058T, 4.17%; A2059G, 51.11%
101	-	AG	101-1	0.25	0.5	S	-	AA	Yes	AA	A2059G, 7.73%
102	+	TA	102-1	≥256	≥256	R	+	AA	Yes	TA	
103	-	TG	103-1	≥256	≥256	R	+	AA	Yes	TA	A2058T, 52.23%; A2059G, 7.42%
104	-	AA	104-1	≤0.25	≤0.25	S	-	AA			
105	-	AA	105-1	0.25	0.5	S	-	AA			
106	+	TA	106-1	≥256	≥256	R	+	AA	Yes	TA	
			106-2	0.25	0.5	S	-	AA			
